# Supplementary material for: Identification of photoperiod-induced specific miRNAs in the adrenal glands of Sunite sheep (Ovis aries)
Source: Front Vet Sci. 2022 Jul 22;9:888207. doi: 10.3389/fvets.2022.888207 (PMC9354845; doi:10.3389/fvets.2022.888207)
Supplement: Supplementary file 1 [file Data_Sheet_1.ZIP › Supplementary file/miRDeep2 file.zip!/miRDeep2 file/Novel_Structure/SPLP42A2/Novel_338.pdf]

Provisional ID	:	NC_019464.2_17147
Score total	:	2.1
Score for star read(s)	:	-1.3
Score for read counts	:	0
Score for mfe	:	1.8
Score for randfold	:	1.6
Score for cons. seed	:	
Total read count	:	200
Mature read count	:	200
Loop read count	:	0
Star read count	:	0

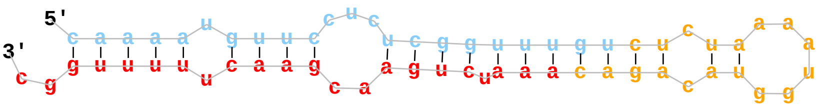

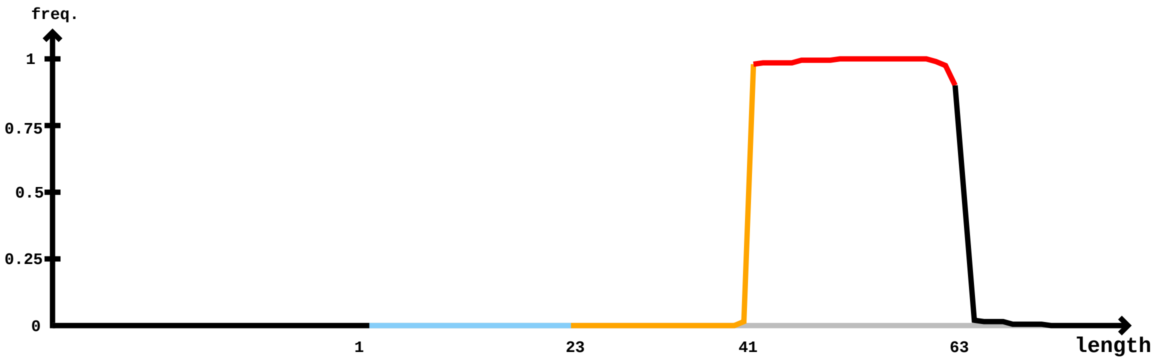

Star   Mature

```
5'- aaacugaccuuuuccauuguauuggguuggccaaaauguuccucucgguuugucucuaaaaugguacagacaaaucugaacgaacuuuuuggccaacccaaacuuccuug   -3'    exp
    ..................((.(((((((((((((((.((((...(((((((((((.((......)).))))))).))))..)))).)))))))))))))))))).......    reads    mm    sample
    ......................................................................caaaucugaacgaacuuuuu....................    1        0     seq
    ......................................................................Aaaaucugaacgaacuuuuugg..................    2        1     seq
    .......................................................................aaaucugaacAaacuuuuu....................    1        1     seq
    .......................................................................aaaucugaacAaacuuuuug...................    3        1     seq
    .......................................................................aaaucugaacAaacuuuuugg..................    13       1     seq
    .......................................................................aaaucugaacAaacuuuuuggc.................    177      1     seq
    ........................................................................aaCcugaacgaacuuuuuggcca...............    1        1     seq
    ............................................................................ugaacgaacuuuuuggccaacc............    2        0     seq
    ................................................................................cgaacuuuuuggccaacccCaa........    1        1     seq
```
